# Spring Diseases

**Published:** 1876-02

**Authors:** 


### SPRING DISEASES.

Any housekeeper would be considered demented who would keep up as fierce a fire on the hearth in the spring as in mid-winter. On the contrary as the days grow warmer, less and less fuel is used, until the fire is not kindled at all. One of the two main objects of eating is to keep the body warm; and it need not be argued that less warmth is required in summer than in winter; but if we eat as heartily as the spring advances as we did in cold weather, we will burn up with fever, because we have made too much heat. The instincts of our nature are perfectly wonderful. To our shame is it, that we not only do not heed them, but oppose them, fight against them with an amazing fatuity. As the warm weather comes on, we are all conscious of a diminution of appetite, and we either begin to apprehend we are about to get sick, or set about stimulating ourselves with tonics, and bitters, and various kinds of teas, with a view to purifying the blood. How many swills of sassafras-tea has the reader taken to that end! No such purification would be needed, if we would follow nature's instincts, and eat only with the inclination she gives us, instead of taking tonics to make us eat more, when we actually require less.

Observant persons have noticed that as spring comes on, there is less relish for meats of all kinds, and we yearn for the early spring vegetables, the "greens," the salads, the spinnage, the radishes, and the like.

